# Health Impact Database Development for Sweeteners and Sweetness Enhancers: The SWEET Project

**DOI:** 10.1111/nbu.70006

**Published:** 2025-04-03

**Authors:** Corey E. Scott, Nikoleta Stamataki, Joanne A. Harrold, Anne Raben, Jason C. G. Halford

**Affiliations:** ^1^ Cargill Core Research and Development Plymouth Minnesota USA; ^2^ Department of Psychology, Institute of Population Health University of Liverpool Liverpool UK; ^3^ Department of Nutrition, Exercise and Sports University of Copenhagen Copenhagen Denmark; ^4^ Clinical Research Steno Diabetes Center Copenhagen Herlev Denmark; ^5^ School of Psychology, Faculty of Medicine & Health University of Leeds Leeds UK

**Keywords:** database, high‐intensity sweeteners, low and non‐caloric sweeteners, non‐nutritive sweeteners, sugar, Sweeteners

## Abstract

Sweeteners and sweetness enhancers (S&SEs) are ingredients used in foods and beverages to reduce sugar while providing the sweetness of sugar with little to no calories. Although S&SEs have global regulatory approval and acceptance, questions remain regarding their overall safety and efficacy. Information on the effects of S&SEs in regard to health and efficacy can be found in randomised controlled trials (RCTs) that exist in peer‐reviewed literature. With the large number of RCT publications on various S&SEs, a need exists to organise and collect each of the published studies in a useful database. Currently, a database containing human clinical information on S&SEs does not exist and so The *SWEET* project has created a publicly available and comprehensive Health Impact Database that includes available human clinical information on sweeteners. This paper describes the process and development of a database that collects comprehensive information on published human clinical studies evaluating S&SEs between the years January 2000 and September 2024. Ovid Medline was used to search for RCT publications from the year 2000 to 2024. The search produced 1538 publications, of which 257 complied with the predetermined eligibility criteria. There was a large variability in the number of studies that fit the inclusion criteria. For example, some S&SEs had numerous studies (i.e., sucralose, *n* = 63 eligible publications) and some S&SEs had no publications that fit the criteria (aspartame‐acesulfame K salt and neohesperidine DC). The Health Impact Database is located at https://sweetproject.eu/HIdatabase and is contained in Microsoft Excel spreadsheets which are organised by health impact criteria. This database will be a useful tool for researchers as it provides comprehensive information on human clinical studies on S&SEs that can be leveraged as a general resource and for systematic reviews and meta‐analyses.

## 1 Introduction

Sweetness and sweetness enhancers (S&SEs) are useful tools to reduce sugars in foods and beverages. S&SEs represent a class of diverse molecules that typically supply the sweetness of sucrose but at much smaller (100–1000 fold) amounts and can be used in foods and beverages to supply sweetness with little to no energy. Common S&SEs include artificial sweeteners such as sucralose, aspartame and acesulfame‐K, and plant‐based sweeteners, such as stevia and monk fruit extracts. Although S&SEs have global regulatory approval, the safety and efficacy of S&SEs against some health outcomes are controversial, and their continued use has been questioned (Rios‐Leyvraz and Montez 2022). Furthermore, there are differences in findings between observational studies and randomised controlled trials (RCTs) (Lohner et al. 2017; Higgins and Mattes 2019; Toews et al. 2019; Andrade et al. 2021; Pang et al. 2021; Rios‐Leyvraz and Montez 2022). Observational studies typically establish correlations or associations over the long term, whereas RCTs, which are considered the gold standard of studies but are generally more short term, are useful in establishing causal relationships and are more relevant to establishing the safety and efficacy of S&SEs. It is therefore important to collect human clinical information on S&SEs in a comprehensive database that can be used as a key point of reference which organises health‐related information from human clinical studies regarding the use of S&SEs in humans. Thus, RCTs which evaluate S&SEs under controlled circumstances are captured in the database rather than observational studies. A similar database has been generated for dietary fibres (Diet‐Related Fibres and Human Health Outcomes Database; Tufts University; Livingston et al. 2016). A need for a database on S&SEs was proposed in 2016 along with an evidence map (Wang et al. 2016); however, as of early 2024, no such database existed.

Therefore, the *SWEET* project aimed to create such a database for use as a reference tool for RCTs evaluating S&SEs and which could also serve as a tool useful for carrying out systematic reviews and meta‐analyses. The Health Impact Database is publicly available and accessible via a web‐based portal at https://sweetproject.eu/HIdatabase and downloadable. A table/spreadsheet format is used for the Health Impact Database and lists the S&SEs corresponding health impact data.

## 2 Methods

### 2.1 Search Strategy

A search strategy was implemented to identify and collect human clinical studies to be included in the database. This included inclusion and exclusion criteria, sweetener types, health outcomes and data organisations into spreadsheets. Ovid Medline (http://ovidsp.ovid.com/) was used to search for adult human clinical trial publications and search terms included S&SEs sucralose, aspartame, acesulfame‐K, saccharin, stevia, steviol glycosides, advantame, neotame, monk fruit extract, mogrosides, neohesperidine DC, aspartame‐acesulfame salt and thaumatin. Commercial brand names, including Truvia, Splenda and Equal, were also searched. Main health outcomes areas (bodyweight, glycaemia, food intake, dental caries etc.) were searched in line with health outcomes in RCTs in recent systematic reviews and meta‐analyses but not typical of longer‐term observation studies (i.e., cancer, kidney disease, mood and neurocognition; Toews et al. 2019; Rios‐Leyvraz and Montez 2022). See Data S1 and S2 for further details. Online searches for publications were conducted between January 2019 and September 2024. The resulting publications were extracted and screened for title and abstract to decide on eligibility. In addition, a recent publication database which included studies S&SEs up to 2014 was screened for additional studies (Wang et al. 2016). Recent citations from systematic reviews and meta‐analyses were used to search for further publications (Toews et al. 2019; Andrade et al. 2021; Pang et al. 2021; Rios‐Leyvraz and Montez 2022).

### 2.2 Human Clinical Study Inclusion and Exclusion Selection Criteria

In order to screen a potentially large number of publications and fit within the budgetary and timing constraints of the *SWEET* project, inclusion and exclusion criteria were determined. The inclusion and exclusion criteria were similar for previously published systematic reviews and meta‐analyses. Inclusion and exclusion criteria used to screen publications for the Health Impact Database are listed in Table 1.

**TABLE 1:** Health Impact Database eligibility—Inclusion and exclusion criteria.

Study eligibility—Inclusion criteria	Study eligibility—Exclusion criteria
Human volunteers Intervention studies (randomised or non‐randomised, controlled) Health status: any Current FDA and/or EFSA‐approved sweeteners Sweeteners used individually or in combinations (separate database for studies that looked at blend/combinations of sweeteners within one treatment) Studies published between 2000 and 2024 Studies published in English Peer‐reviewed published papers	RCTs published before 2000 Animal and in vitro studies Prospective cohort studies Case–control studies Cross‐sectional studies Case reports Systematic Reviews and Meta‐analyses Studies which used S&SEs as a control. Regulatory dossiers Toxicological reports Grey literature White papers

Abbreviations: EFSA, European Food Safety Authority; FDA, US Food and Drug Administration; RCT, randomised controlled trials; S&SEs, sweeteners and sweetness enhancers.

## 3 Results

### 3.1 Search Findings by Specific Sweetener

**FIGURE 1:**
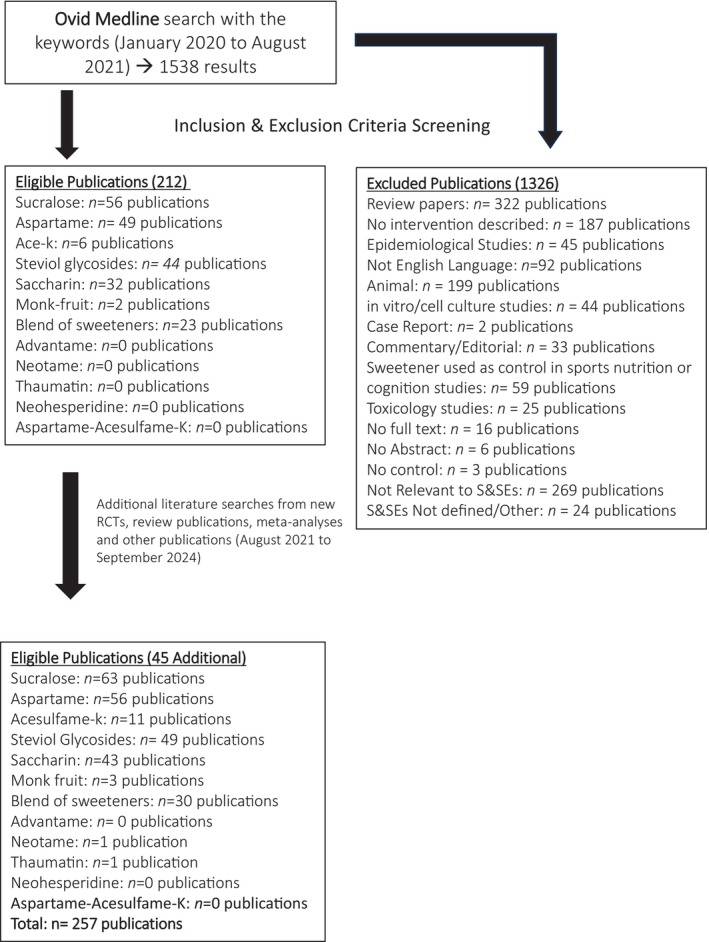
Search strategy for eligible publications. Searches from January 2020 to August 2021 coincided with the timeline of THE SWEET Project deliverable deadline. Additional searches were performed between August 2021 and September 2024. Publications listed as Not Relevant includes publications that discussed S&SES but did not specifically evaluate S&SEs. S&SEs Not Defined/Other includes publications that used S&SEs that were out of scope such as polyols or other carbohydrates. The list of 1326 excluded publications also includes 405 publications published before the year 2000.

The Ovid Medline search (January 2020–August 2021) and search of additional publications (August 2021–September 2024) resulted in 1538 total publications. After excluding publications based on the inclusion/exclusion eligibility criteria, a total of 257 publications were included in the Health Impact Database (see Figure 1). The number of publications for each sweetener that met the inclusion/exclusion criteria is listed in Table 2. There was a large variability in the number of studies that fit the inclusion criteria. For example, some S&SEs were represented in a relatively large number of studies (i.e., sucralose, *n* = 63 eligible publications) whereas other sweeteners were not included in studies that fit the criteria (aspartame‐acesulfame‐K salt and neohesperidine DC). Health outcome measures are listed in Table 3 and the largest health outcome measures in the eligible studies are glucose homeostasis, followed by energy intake, bodyweight and gut hormones. The least reported health outcome measures are oral health and gut microbiome. The S&SEs blends comprised 30 of the eligible publications and were largely blends of artificial S&SEs aspartame and acesulfame‐K, sucralose & acesulfame‐K, and also plant‐based S&SEs stevia & monk fruit and stevia & thaumatin.

**TABLE 2:** List of eligible sweeteners and sweetness enhancers (S&SEs) publications leveraged for the Health Impact Database.

S&SEs type	Number of eligible publications
Sucralose	63
Aspartame	56
Saccharin	43
Blend of sweeteners^a^	30
Steviol glycosides	49
Unspecified sweeteners^b^	8
Acesulfame‐K	11
Monk fruit extract	3
Advantame	0
Thaumatin	1
Neotame	1
Neohesperidine DC	0
Aspartame‐acesulfame‐K	0
Total included in database	**257**

*Note:* Eligible publications from search strategy for each S&SEs based on inclusion and exclusion criteria in peer reviewed journals from January 2000 to September 2024. Number of studies are accurate as of September 2024.

^a^ Blends of sweeteners are captured on each respective database spreadsheet. For example, if a blend contained stevia and monk fruit extract, it appears in both stevia and monk fruit extract spreadsheets.

^b^ Unspecified sweeteners are not included in the databases and their respective references are captured in Data S1 and S2.

**TABLE 3:** Health outcome measures and metrics used for the Health Impact Database.

Health outcome measure	Metrics	Number of studies measuring each health outcome
Bodyweight	Primary: Bodyweight, BMI Secondary: body composition (body fat, fat‐free mass)	31
Energy intake/appetite	Subjective appetite ratings Food/energy intake (short term), energy intake (long‐term interventions)	54
Glucose homeostasis	Fasting and/or postprandial glucose, insulin, C‐peptide, glucagon	89
Behavioural and/or cognitive	Memory, cognitive tasks, food cue tasks	10
Gut hormones	GLP‐1, GIP, PYY, ghrelin, CCK	28
Blood lipids	Total cholesterol, LDL‐c, HDL‐c, VLDL‐c, Triglycerides	22
Blood pressure	Systolic blood pressure, diastolic blood pressure	13
Brain function	fMRI, EEG	14
Oral health	Dental caries, saliva pH, plaque formation	10
Gut microbiota	Composition of gut microbiome, SCFA profile	10
Other	Liver & Kidney function markers: GGT, ALAT, ASAT, urea, creatinine, haematological parameters, blood chemistries, hormonal levels (other than appetite hormones), gastric emptying	12

*Note:* Various health outcome measurements and metrics from S&SEs RCTs in peer‐reviewed publications that were used for The Health Impact Database in line with outcomes from similar published systematic reviews and meta‐analyses. Number of studies is accurate as of September 2024 and many studies measured more than one health outcome.

Abbreviations: ALAT, alanine transaminase; ASAT, aspartate transaminase; BMI, body mass index; CCK, cholecystokinin; EEG, electroencephalogram; fMRI, functional magnetic resonance imaging; GGT, gamma glutamyl transferase; GIP, gastric inhibitory peptide; GLP‐1, glucagon like peptide; HDL‐c, high density lipoprotein; LDL‐c, low‐density lipoprotein; PYY, peptide YY; SCF, short chain fatty acid; VLDL‐c, very low‐density lipoprotein.

### 3.2 Database Structure, Location and Usage

The Health Impact Database is located at https://sweetproject.eu/HIdatabase and it organises comprehensive data from adult human clinical studies that are collected per type of S&SEs in Excel workbook spreadsheets. The database is organised by health outcome measures (each worksheet within a particular workbook corresponds to one health outcome). Each study is organised by publication date and includes further information, such as the number of volunteers, sweetener dose, trial duration and outcomes. S&SEs, which are used in human clinical studies, are limited to sucralose, aspartame, stevia, monk fruit, acesulfame‐K, advantame and saccharin, as S&SEs aspartame‐acesulfame‐K salt and neohesperidine DC had no eligible publications. Studies that assessed more than one S&SE are included in each respective database. For example, if one study evaluated the effects of stevia and aspartame on food intake, this study was included in both the stevia and the aspartame databases. Studies which evaluated more than one of the outcomes from Table 3 were included in all the relevant categories. For example, a study that assessed glucose homeostasis and food intake was included in both spreadsheets for food intake and glucose homeostasis. Within the website link, there are also the Guidelines, Terms of Use and Citation, and users are encouraged to review and comply with the Guidelines and Terms of Use of the Health Impact Database.

## 4 Discussion and Conclusion

There are a large number of publications based on human clinical studies evaluating S&SEs, and it is important to collect these studies into a comprehensive database for use as a reference and to facilitate information for systematic reviews and meta‐analyses. The *SWEET* project Health Impact Database is the first database of its kind to collect, organise and capture comprehensive information on several S&SEs from human clinical studies from the years 2000 to 2024. In generating the Health Impact Database, we observed diversity in the number of eligible publications, such as S&SEs sucralose and aspartame having many eligible publications and S&SEs neotame, thaumatin, neohesperidine DC and aspartame‐acesulfame‐K having few or no eligible publications. Regarding health outcomes, the most studied outcomes were glucose homeostasis, energy intake and bodyweight, with the least being oral health and gut microbiome. These outcomes are in agreement with S&SEs intended use, as they are largely used to replace or reduce sugar, so it is important to understand how S&SEs perform relative to sugars in regards to glucose homeostasis, bodyweight management, energy intake and gut hormones. This database will be useful for researchers who endeavour to evaluate human clinical data from various S&SEs studies and can be leveraged as a point of reference to identify key clinical studies evaluating S&SEs in several categories in various human populations.

## 5 Limitations

The Health Impact Database largely includes information on health outcomes from human clinical studies undertaken between the years 2000 and 2024. A decision was made within the *SWEET* project to include studies from the year 2000 mostly due to budgetary and time constraints in generating the database within the project parameters. Studies have been identified from before the year 2000, and they largely include studies on aspartame and saccharin. Aspartame had a total of 91 publications that fit the eligibility criteria; however, only 56 publications were published after the year 2000. Many eligible saccharin studies were published before the year 2000 and few between our inclusion time of the years 2000–2024. Thus, a decision was made to include eligible saccharin studies before the year 2000 in order to capture saccharin in the database. As the database is updated, new studies and studies before the year 2000 can be included. Only one database (Ovid Medline) was used in the search, and it is likely The Health Impact Database may not include all eligible S&SEs studies. Observational studies were not in scope for the *SWEET* project and were not captured in the database. Study quality was not addressed as this was outside of the scope of the *SWEET* project, which was rather to capture the information from S&SEs RCTs in a database. The authors recommend that future systematic reviews or meta‐analyses assess study quality from the original publications. Some sweeteners did not have publications which met the inclusion criteria (neohesperidine DC, advantame, aspartame‐acesulfame‐K) and are not included in the database. Sweeteners that were not specified are not captured in the database. Human clinical data can exist in regulatory dossiers or similar types of literature which may or may not be publicly available. The scope of the *SWEET* project was to solely evaluate published and publicly available literature, and given these noted limitations, this database will be useful as it organises comprehensive information on health outcomes from S&SEs RCTs.

## Conflicts of Interest

C.E.S. and N.S. were paid employees of Cargill Inc. and Cargill, B.V. during the preparation of the Health Impact Database. J.C.G.H. and J.A.H. have received project funds from the American Beverage Association. A.R. has received honoraria from Nestlé, Unilever and the International Sweeteners Association.

## Supporting information


**Data S1.** References to unspecified sweeteners.

**Data S2.** Ovid Medline search results.

## Data Availability

The data from The Health Impact Database are currently available on the Project Sweet Website at https://sweetproject.eu/HIdatabase.
