# Supplementary material for: Health Impact Database Development for Sweeteners and Sweetness Enhancers: The SWEET Project
Source: Nutr Bull. 2025 Apr 3;50(2):340–4. doi: 10.1111/nbu.70006 (PMC12147055; doi:10.1111/nbu.70006)
Supplement: Supplementary file 1 — Data S1. References to unspecified sweeteners. [file NBU-50-340-s001.docx]

**Supplemental Material**

**Review Publications Screened For The Health Impact Database**

Review Publications Screened:

Andrade, Lesley et al. “Low-calorie sweeteners and human health: a rapid review of systematic reviews.” Nutrition reviews vol. 79,10 (2021): 1145-1164. doi:10.1093/nutrit/nuaa123

Rios-Leyvraz, Magali & Montez, Jason. (2022). Health effects of the use of non-sugar sweeteners: a systematic review and meta-analysis.

Lee, Han Youl et al. “Effects of Unsweetened Preloads and Preloads Sweetened with Caloric or Low-/No-Calorie Sweeteners on Subsequent Energy Intakes: A Systematic Review and Meta-Analysis of Controlled Human Intervention Studies.” Advances in nutrition (Bethesda, Md.) vol. 12,4 (2021): 1481-1499. doi:10.1093/advances/nmaa157

Pang, Michelle D et al. “The Impact of Artificial Sweeteners on Body Weight Control and Glucose Homeostasis.” Frontiers in nutrition vol. 7 598340. 7 Jan. 2021, doi:10.3389/fnut.2020.598340

Rogers, Peter J, and Katherine M Appleton. “The effects of low-calorie sweeteners on energy intake and body weight: a systematic review and meta-analyses of sustained intervention studies.” International journal of obesity (2005) vol. 45,3 (2021): 464-478. doi:10.1038/s41366-020-00704-2

Greyling, Arno et al. “Acute glycemic and insulinemic effects of low-energy sweeteners: a systematic review and meta-analysis of randomized controlled trials.” The American journal of clinical nutrition vol. 112,4 (2020): 1002-1014. doi:10.1093/ajcn/nqaa167

Yeung, Andy Wai Kan, and Natalie Sui Miu Wong. “How Does Our Brain Process Sugars and Non-Nutritive Sweeteners Differently: A Systematic Review on Functional Magnetic Resonance Imaging Studies.” Nutrients vol. 12,10 3010. 30 Sep. 2020, doi:10.3390/nu12103010

Bueno-Hernández, N et al. “Review of the scientific evidence and technical opinion on noncaloric sweetener consumption in gastrointestinal diseases.” “Revisión de la evidencia científica y opinión técnica sobre el consumo de edulcorantes no calóricos en enfermedades gastrointestinales.” Revista de gastroenterologia de Mexico (English) vol. 84,4 (2019): 492-510. doi:10.1016/j.rgmx.2019.08.001

Nichol, Alexander D et al. “Glycemic impact of non-nutritive sweeteners: a systematic review and meta-analysis of randomized controlled trials.” European journal of clinical nutrition vol. 72,6 (2018): 796-804. doi:10.1038/s41430-018-0170-6

Santos, Natalia Cardoso et al. “Metabolic effects of aspartame in adulthood: A systematic review and meta-analysis of randomized clinical trials.” Critical reviews in food science and nutrition vol. 58,12 (2018): 2068-2081. doi:10.1080/10408398.2017.1304358

Bundgaard Anker CC, Rafiq S, Jeppesen PB. Effect of Steviol Glycosides on Human Health with Emphasis on Type 2 Diabetic Biomarkers: A Systematic Review and Meta-Analysis of Randomized Controlled Trials. Nutrients. 2019 Aug 21;11(9):1965. doi: 10.3390/nu11091965. PMID: 31438580; PMCID: PMC6770957.

Tan, Sze-Yen, and Robin M Tucker. “Sweet Taste as a Predictor of Dietary Intake: A Systematic Review.” Nutrients vol. 11,1 94. 5 Jan. 2019, doi:10.3390/nu11010094

Tucker, Robin M, and Sze-Yen Tan. “Do non-nutritive sweeteners influence acute glucose homeostasis in humans? A systematic review.” Physiology & behavior vol. 182 (2017): 17-26. doi:10.1016/j.physbeh.2017.09.016

**Supplemental Material**

**Publications That Did Not Specify Sweeteners**

Ebbeling, Cara B et al. “A randomized trial of sugar-sweetened beverages and adolescent body weight.” The New England journal of medicine vol. 367,15 (2012): 1407-16. doi:10.1056/NEJMoa1203388

Tate, Deborah F et al. “Replacing caloric beverages with water or diet beverages for weight loss in adults: main results of the Choose Healthy Options Consciously Everyday (CHOICE) randomized clinical trial.” The American journal of clinical nutrition vol. 95,3 (2012): 555-63. doi:10.3945/ajcn.111.026278

Anne Raben, BenteK. Møller, Anne Flint, TatjanaH. Vasilaras, A. Christina

Møller, Jens Juul Holst & Arne Astrup (2011) Increased postprandial glycaemia, insulinemia,

and lipidemia after 10 weeks’ sucrose-rich diet compared to an artificially sweetened

diet: a randomised controlled trial, Food & Nutrition Research, 55:1, 5961, DOI: 10.3402/

fnr.v55i0.5961

MJM Munsters and WHM Saris

Annals of Nutrition &Metabolism 2010;57(2):116-23

Weijzen PLG, Smeets PAM, de Graaf C. Sip size of orangeade: effects on intake and sensory-specific satiation. British Journal of Nutrition. 2009;102(7):1091-1097. doi:10.1017/S000711450932574X

Ilse Skokan, P Christian Endler, Beatrix Wulkersdorfer, Dieter Magometschnigg and Heinz Spranger Influence of Artificial Sweetener on Human Blood Glucose ConcentrationBrief Research Report

TheScientificWorldJOURNAL, (2007) 7, 1618–1621 TSW Holistic Health & Medicine

ISSN 1537-744X; DOI 10.1100/tsw.2007.228

Ebbeling, Cara B et al. “Effects of decreasing sugar-sweetened beverage consumption on body weight in adolescents: a randomized, controlled pilot study.” Pediatrics vol. 117,3 (2006): 673-80. doi:10.1542/peds.2005-0983

S.H.A Holt, L Cobiac, N.E Beaumont-Smith, K Easton, D.J Best, Dietary habits and the perception and liking of sweetness among Australian and Malaysian students: A cross-cultural study,Food Quality and Preference,Volume 11, Issue 4,2000, Pages 299-312,ISSN 0950-3293,
